# Injuries and Diseases of the Mouth and Their Treatment

**Published:** 1893-05

**Authors:** J. Adams Bishop

**Affiliations:** New York


### International Dental Journal.

Vol. XIV. May, 1893. No. 5.

### Original Communications.[1]

### INJURIES AND DISEASES OF THE MOUTH AND THEIR TREATMENT.[2]

BY DR. J. ADAMS BISHOP, NEW YORK.

Mr. President and Gentlemen of the Odontological Society,—According to the monthly notice of your Executive Committee, I am to read this evening a paper on the "Injuries and Diseases of the Mouth and their Treatment."

Such treatment is either surgical or non-surgical, or mechanical. Surgeons and dentists have to seek the assistance of one another to effect cures. Sometimes the dentist has to prepare the way for the surgeon's knife, and in other cases the dentist has to aid the surgeon by a mechanical support. Opportunities of this kind are constantly coming to our hands, and we should be thoroughly familiar with the methods of treatment proper in each case, or else we should turn the patient over to some one who is.

The cases I shall to-night speak of particularly have never, to my knowledge, been presented to a dental society nor put forth in a dental publication, but were so important that the surgeons in charge have reported them before the Academy of Medicine, the

[1] The editor and publishers are not responsible for the views of authors of papers published in this department, nor for any claim to novelty, or otherwise, that may be made by them. No papers will be received for this department that have appeared in any other journal published in the country.

[2] Read before the New York Odontological Society, February 21, 1893.

New York State Medical Society, and the National Medical Society, and these reports can be found in their publications and in the "Medical and Surgical History of the Rebellion," Part First. The casts are also to be found at the Anatomical Museum at Washington.

I will present first a case of partial reconstruction of the face, found in "Buck's Reparative Surgery," 1876. The patient was Carleton Burgan, aged twenty years, a volunteer in one of the Maryland regiments. His history, previous to his admission to the New York Hospital, was furnished by Robert F. Weir, M.D., assistant surgeon United States Army, in charge of the Army General Hospital at Frederick, Maryland (where Burgan had been a patient before coming to this city).

"He was taken sick June 5, 1862, with rheumatic pains, due to exposure to wet and cold. During the next month he was under treatment at the army hospital for typhoid fever, and in the middle of August, when his general condition appeared to be improving, gangrene set in, and, beginning with a small black slough, attended with fetor, on the gum at the foot of the first right upper bicuspid tooth, rapidly extended until it had involved and destroyed the right half of the upper lip, the adjacent portion of the cheek, and the ala of the nose, and denuded the entire superior maxillary bone. By the middle of October contraction of the healing parts had taken place to such a degree that the hole in the face was diminished nearly one-half in size. On the 23d of December, Burgan was discharged from the hospital in Frederick and from the United States service to go to New York."

On December 31, 1862, when admitted into the New York Hospital (as a patient in the hands of Dr. Gurdon S. Buck, surgeon to the New York Hospital and a member of the Academy of Medicine), his general health was good and continued so. The condition of his face was as follows:

"The right eye was destroyed and sunken; the right half of the upper lip, the right ala of the nose, and the adjacent portion of the cheek, besides the entire right superior maxillary bone, were gone, leaving an extensive opening directly into the cavity of the mouth and right nasal fossa. The margin of the opening, which was everywhere cicatrized, consisted below of the border of the lower lip which was prolonged on the right side, obliquely upwards and outwards, and adhered to the malar bone at a point where the superior maxilla had separated from it. From this point, which corresponds with the middle of the cheek, the margin of the opening extended upwards and inwards in a curved direction to the side of the nose, approaching within a finger's breadth of the inner canthus of the eye, continuing thence along the ridge of the nose to its tip and a little to the right of the median line. The columna nasi being destroyed, the lower edge of the left ala and the rounded margin of the left half of the lip bounded the opening in this direction.

"The middle incisor of the left superior maxilla was wanting; with the exception of this and one molar, the teeth of that side were complete. The lining membrane of the cavity had everywhere a remarkably healthy aspect. The velum still retaining its bony support, performed its functions in deglutition undisturbed. The patient's speech, however, was very indistinct, and resembled that of a person with a bad cleft palate.

"In devising a plan for the restoration of this extensive destruction of parts, it was judged indispensable, as a prerequisite to any surgical operation, that an artificial substitute should be adapted to the cavity of the mouth, to supply the place of the right maxillary bone and afford a solid support to the soft parts that would be required to be transposed for the reconstruction of the mouth and the closure of the cheek and nostril.

"Dr. Thomas B. Gunning, an eminent dentist residing at No. 41 East Twenty-first Street, to whom the case was submitted, generously undertook the execution of this delicate and difficult work. The result, which he achieved after patient and persevering labor, displayed remarkable ingenuity and skill, and cannot fail to elicit universal admiration. The fixtures which he adapted were made of vulcanite, and consisted of two principal pieces. The upper piece being first introduced, occupied the nasal fossa and filled out the right half of the nose. It was hollow, and open in front and behind for the free passage of air. The lower piece formed an artificial palate, covering the entire bony portion of the roof of the mouth, and supplying the right half of the dental arch with the teeth belonging to it. Its left margin took support from the existing teeth of that side, some of which it embraced. The surfaces of both these pieces, where they come in contact with the walls of the nasal and buccal cavities, were channelled with furrowed lines to facilitate the flow of the secretions back into the fauces. Their accurate adjustment to each other and to the walls of the cavities which they occupied, permitted them to be worn without causing the least irritation. A third supplementary piece completed the arrangement. It capped the molar teeth of the lower jaw on the right side, and was connected with the opposite portion of the palate piece above by a spiral wire spring. The improvement of the speech produced by these fixtures, as well as the increased facility of mastication afforded by them, were results highly gratifying to the patient and satisfactory to the surgeon. The material of which they were constructed was admirably adapted, by its light and indestructible nature, to its use. After the patient had worn them constantly for more than two weeks, to test and become habituated to them,—he himself removing and replacing them for the purpose of cleanliness with the greatest facility,—it was decided to perform the first operation on the 26th of March.

"This took place at the New York Hospital in the presence of a large number of physicians. Four operations followed, of rhinoplastic surgery, on April 23, June 18, August 8, and October 27, 1863.

"Within six days after the final operation the sutures were all removed, and the adhesion was perfect. A great improvement in appearance was thus accomplished." [1]

I was present at all of the operations at the hospital, assisted in taking all the impressions, made all the rubber appliances and the casts exhibited here to-night. These appliances, though non-surgical, were of great assistance to the surgeon in giving back to this man the lost speech, mastication, and deglutition,—no small items when considered with his improved appearance. I visited him in Baltimore ten years after this transformation, and had the satisfaction of seeing the same plate in use that he wore when discharged from the hospital.[2]

Dr. Buck brought before a meeting of the Academy of Medicine, on February 7, 1866, the following case:

"Egbert Hewett was wounded, on the 19th of September, 1864, while under Sheridan, at Winchester, Va., by a fragment of shell, which came from the left and struck his mouth. The upper and lower front teeth were carried away, the right cheek was laid open from the mouth to the angle of the jaw, and the lower jaw was fractured at the symphysis. The teeth were replaced on plates in December, 1865; auto-plastic surgery completed the operation, and the patient was discharged January 19, 1866."

This case happened to come under my personal supervision, by my appointment to the staff of St. Luke's Hospital, through the courtesy of Dr. Buck.

---

1 "Buck's Reparative Surgery," Appleton, 1876.

2 Since reading this paper I have again had the satisfaction of seeing this man in Baltimore, and found him still using the same plate, and his face in a good, healthy condition. It proves what good blood he had, that he has outlived all the surgeons who watched these operations.—March 10, 1893.

FRACTURES AND THEIR TREATMENT.

In fractures or injuries presented to us, there are two things to be carefully and thoroughly considered: First, a correct diagnosis must be made to discover the extent and condition of the injury or fracture, in order to set the bones in their normal position, which is handy work for a skilful surgeon or an anatomist; Second, to see what mechanical appliances can be adopted to hold the parts steadily in juxtaposition, so that there may be a reunion by the process of nature, which always comes to our aid whenever and wherever she can.

It is no reflection upon the most skilful and distinguished surgeons to say that they ought, in many cases, to seek the aid of the dentist and his mechanisms to supplement their services in the interest of their patients; for he (the dentist) has experience in mechanical structures, which they (the surgeons) cannot be supposed to readily furnish.

In 1852 the Nelson Goodyear discovery of the process of vulcanizing caoutchouc came to the aid of dentistry. Its value to our profession cannot be over-estimated; and here we first had the opportunity to introduce perfectly interdental splints of vulcanite, which hold the parts so comfortably and firmly as to supersede all appliances of bandages, etc.; not only that, but it sometimes saves the patient from additional trouble, such as abscesses and exfoliations, which often in the past complicated cases treated by the old method of bandaging.

During a long association with the late Dr. Thomas B. Gunning, who was the first to use interdental splints (even before he was called in that historic case of Secretary Seward), I assisted in all his cases, both in private and hospital practice. The late Dr. Gunning is entitled to great respect for his skill and perseverance in whatever he undertook in dentistry, but his uncompromising attitude in the presence of such eminent surgeons as Surgeon-General Barnes and the medical directors of the army and navy was uncalled for. There was neither courtesy nor dignity in his consultation with them, during the treatment of Secretary Seward, in condemning the use of bandages which they used, and treating them as if they were incompetent practitioners or quacks. It was such conduct on the part of Dr. Gunning that deprived our profession of its proper credit, for, when they made their published reports, they studiously avoided all mention of him and his appliance, and no one could blame them.

CASE I.—"By an explosion on a Spanish man-of-war, while firing a salute in our harbor, in 1860, one of the men received the full force of the explosion, and was taken to the U.S.N. Hospital, Brooklyn, where Surgeons Franklin and Gihon were in charge. I have here the casts representing his injuries.[1] They consisted of a comminuted fracture of the lower jaw at the symphysis, the teeth of both jaws much shattered, with face severely burned and lacerated.

"The case had been carefully treated for over four months without producing any union, when, by the advice of Surgeon Bache, Director of the Naval Laboratory, Dr. Gunning was requested to treat it. The jaw was then contracting from loss of bone, and pieces were coming out through the chin. He applied a hard, vulcanized rubber splint, which inclosed the remaining teeth and gum of the lower jaw, its upper surface fitting well over the teeth above, except in front, where it was trimmed down to allow food to pass between the remnants of the superior incisors. The splint was fastened to the lower jaw by screws passing into a broken tooth on each side. The jaw was held up by starched muslin, moulded to a cast of the parts, in repeated folds, until a line in thickness. This reached to the zygomas, and was kept up by a band passing over the head. The splint was applied February 12, 1861. Fragments of the bone came away for some time after, but the splint was not removed during the treatment. The jaw united well by the middle of May, and the man was discharged."

You can get but a faint idea from this report of the labor over this poor man; sick, tired out, with a fracture of the lower jaw of over four months' standing, every part of the face tender to the most gentle touch. And what was the condition on getting the mouth open for correct diagnosis? Anything but inviting. It was foul and slimy with pus, fetor, etc. Surgeons of the hospital had exhausted every method at their command. When Dr. Gunning was called into the case, with me as his assistant, the process of treatment was commenced inside the mouth. It took much time to manipulate the parts of the mouth, to open it sufficiently for cleansing, and then to take the impressions. The external bandages were of no use to us, but, as we could use vulcanite, we soon saw that we could make a cap of it which would help greatly when fastened. We were ten days, trying with many devices, before we thought of using screws to hold it. When they were made and inserted we felt that something had been accomplished; and,

---

[1] New York Academy of Medicine, October, 1863, and *Dental Cosmos*, 1863.

as we watched it from day to day, we found they did not fail us, and we saw a most wonderful improvement going on. He was sent home with this splint in his mouth. This is the first interdental splint ever made of vulcanite, and it opened up a new treatment for the lower jaw.

The medical men of the Navy Hospital and Laboratory were the first to appreciate its benefit, and, as an outgrowth of the case, Dr. Gunning was called to Washington by Surgeon Bache of the Hospital.

Case II.—"Dr. Gunning himself received a compound fracture in his own jaw between the right canine and lateral incisor teeth, on November 4, 1862, through his horse falling under him. The bone was much displaced, and two incisor teeth loosened. He set the bone, and it was held by strong, well-stretched silk, inclosing three incisors, the right canine, and the first bicuspid. This immediately stopped the bleeding and held the bone firmly. A vulcanite splint was applied thirteen hours after the injury. It inclosed all the lower teeth, and was fastened by gold screws to the first molars. It held the fragments so well that he was able to attend patients in the afternoon, and continued so to do subsequently. The gum united by first intention, and the pain and swelling, which were very great in the external parts, diminished rapidly. On November 28 the splint was removed, and good but flexible union found. It was again fastened on, but, after seven days, was worn without the screws, and removed daily. The jaw grew strong, the teeth firm, and the splint was left off January 1, 1863, but worn at night until February 1. The jaw was used in eating, talking, etc., throughout the treatment."

The case was presented to the New York Academy of Medicine, January 7, 1863, by Dr. A. L. Sands. He refers to a conversation had with Dr. Gunning, in which the latter outlined his plan for securing a broken jaw, and says he had promised, in case of meeting with such an injury, to try it, and comments on Dr. Gunning being one of the first to receive the benefit of his own ingenuity.

In the discussion that followed the reading of the paper, Dr. Alex. N. Stephens, Professor Emeritus of Clinical Surgery in the College of Physicians and Surgeons, said,—

"I look upon this splint as far beyond anything in the way of treating fractures of the lower jaw that has ever been discovered, and I congratulate the Academy on this discovery, for I fully believe it to be of such importance that it will last forever."

The splint was shown the Medical Society of the State of New York in February, 1863.

I wish to present to you a few additional facts about this case of Dr. Gunning's.

When he came into his house, he said, "I have broken my jaw." "It can't be so," I said to him. He took hold of each side of his fracture and pulled it apart so that I could pass my finger through it.

Dr. Sands was sent for, also Dr. Hosuck, who lived very near. Dr. Hosuck cared for his face and prescribed for him rest and quiet, and said in a few days he would care for the bone. Dr. Gunning thanked him and said he would be glad to see him, but would treat his fracture himself. He then said to me, "Whom can you get to help you to make my splint, so that it can be placed on the jaw to-morrow morning?" I availed myself of the kind services of Dr. —— (by request, his name is omitted). He took the impression about eight P.M., remaining all night to help make the splint, and we were able the next morning at nine to place it in the mouth. It was a very strange coincidence that Dr. Gunning should receive such a fracture, eighteen months after the Navy Hospital case, to enable him to receive and prove his own medicine or method. It proves the great value of this treatment of a fracture that he never lost an hour from his office. How different it would have been had it been treated under the old method of bandaging! This was the second interdental splint of vulcanite ever used.

Case III.—"G. B., aged forty-five years. His jaw was fractured by a blow through the socket of the right second bicuspid, June 5, 1863. There was a displacement of the back fragment inward and forward. The patient could not lie down, but slept in a chair holding the jaw, as the surgeons could not keep the fragments in place. The fracture commenced inside the first bicuspid tooth, and passed backward and outward through the socket of the second, and downward also at the expense of the back fragment. As the loosened bicuspid had been extracted, instead of being kept in place, there was nothing to prevent the back fragment from sliding inwards and over the front one and impinging on the inferior dental nerve. The splint was worn until September 3. The jaw was allowed its natural motions throughout the treatment."

This splint was presented to the New York Academy of Medicine in October, 1863. Dr. Gunning received the thanks of the Academy, accompanied by a request to report further when he should have completed the splint which he considered best adapted for general use.

This case (No. 3) was a very difficult one. Mr. B. lived on Washington Square, north. He received the fracture at ten P.M., while out for a stroll on the square, nearly opposite his house, by being hit with a slung-shot. His physician (a member of the Academy of Medicine at the time Dr. Sands reported this interdental splint, five months before), and two other physicians were called in, and yet the only thing they did for the patient was to apply external bandages and tell him he would have to sit in his chair and keep his mouth as still as he could until nature had affected a union.

These physicians had entirely forgotton, if they had ever heard of, Dr. Sands's paper and Dr. Stephens's remarks upon it; and it was left for a lady friend of Mrs. B. to tell her of Dr. Gunning and his method, and the experience he had had with his own mouth.

This fracture being at the inferior dental nerve, every movement, however slight, would cause him great pain, and his sleep was greatly disturbed even while in a sitting posture, for the moment sleep came the hold of the muscles was relaxed, and, the jaw falling, he would be awakened instantly by the pain. Some days passed before the splint was fastened to his mouth, but this so controlled the muscles that on returning home he lay down on his bed and had his first good sleep for ten days.

This is my experience with this patient, whom I saw every day.

CASE IV.—" J. Q., twenty-five years of age, had his jaw broken by being thrown from a cart on December 29, 1863. On the same day he called in a physician, who tied the teeth together and sewed up a deep gash over the left masseter muscle. The ligature did not permanently control the fracture; the teeth became very loose and the front of the jaw was drawn back inside of the left fragment. Patient went into the Bellevue Hospital on January 9, 1864. The left lateral incisor, loosened by the accident, having been extracted, attempts were made to hold the jaw in place by passing wire around the teeth, but without success. On January 14 the patient was brought to Dr. Gunning's office. He found the jaw fractured through the socket of the left lateral incisor, slanting towards the symphysis as it descends. The gum was red and painful; there was great tenderness under the jaw and upon the ramus, which was also supposed to be fractured. On January 15 he applied a vulcanite splint without screws or any fastening. It held the fragments in place, and the patient experienced great relief. On February 13 he took off the splint temporarily; no displacement followed, but the union was very soft. After this he removed the splint and examined the parts weekly. On March 9 the wound was healed.

On April 9 the union was strong, but it was deemed advisable to wear the splint longer on account of the canine tooth which was not yet firm. The jaw articulated with the upper, and the upper and lower teeth fitted against each other well. On May 1 the splint was dispensed with."

Case V.—"Mary Ann D., twenty-nine years of age, was found in a state of insensibility February 12, 1864, and sent to the Bellevue Hospital the next morning. She remained unconscious until the 16th. On February 17 Dr. R. B. Brownell spoke to Dr. Gunning of her broken jaw, but said nothing could be done to it at present, as her head and face were so terribly swollen. On February 21 Dr. Gunning saw the patient at the hospital, and found her lower jaw broken on the right side, commencing half an inch back of the canine tooth and passing downward at the expense of the back fragment. There had been no teeth back of the canine for some time, and, the gum being torn, the back fragment rode over the front, with its point sticking out sharp and bare for three-eighths of an inch in the direction of the symphysis. On February 22 the patient was taken to Dr. Gunning's office. The swellings on the face had lessened somewhat, but were still undiminished in the gum around the fracture. On the left side there were no teeth back of the bicuspid, and the gum was sound and healthy, but indented by the upper wisdom tooth which had been pressing into it since the accident, previous to which it had not done so. This condition of the parts induced him to examine the left ramus carefully, and he found great play of its upper back portion, especially inward; but the only displacement, when at rest, was upward and forward, and this to no great extent, as it was checked by the upper wisdom tooth. He finally concluded that a fracture existed in the neck of the condyle, passing downward and backward, thus allowing the muscles to draw the bone upward and forward. The lower jaw contained only the four front teeth, the two canines, and the first left bicuspid; the gum back of these was free from roots, except that of the right wisdom tooth, which still remained, but was decayed close down. The upper jaw had been without the eight teeth forward of the second bicuspids for some time; of the other eight, seven still remained, the right second molar only having been extracted. To set the jaw the right fragment was put in the best position that could be obtained with the fingers, assisted by a stout piece of silk passing round the left canine. A jack-screw with a collar fitting against the root of the wisdom tooth in right fragment, and the other end bearing on the gum between the left lateral incisor and canine teeth, was then screwed out until the extension was sufficient to allow the fractured bone to come into proper position. The end of the long or forward fragment was then held up, and an impression in soft wax taken of all the teeth and gum as far back as the ramus on each side. Care was taken to put the bone in place at the neck of the condyle, while the bite was obtained. On February 28 he applied the splint. On March 2 the patient brought a request from the surgeon in charge of her case at the hospital that Dr. Gunning would screw the splint fast to the teeth. The gum had grown over the point of the bone; it was now, therefore, only a simple fracture. He screwed the splint fast. On March 14 the patient was in good spirits and quite comfortable. She wanted to leave the hospital and go to work. She made no complaint as to the teeth. On March 26, at Dr. Gunning's request, the patient was discharged from the hospital, but still wearing her splint. On April 8 the splint was removed and a good union found. The splint was worn just forty days, but the patient had a good constitution, and the bone united rapidly. On June 7, 1865, the patient sent word to Dr. Gunning that the jaw was all right."

Cases IV. and V., as here reported, were treated in Bellevue Hospital on December 29, 1863, and February 12, 1864, respectively. I have no comments to make on these, as they are fully reported.

Case VI.—Wm. H. Seward, President Lincoln's Secretary of State, sustained a fracture of the lower jaw, as we all know. The injury was caused by falling from a carriage April 4, 1865. Unsuccessful attempts had been made to hold the jaw in place by bandages, and also with ligatures on the teeth, by the surgeons first called to the case. On the 14th, the date of that awful tragedy in Washington, when Lincoln was killed, the assassin Payne forced himself past Mr. Seward's son, nearly killing him at the door defending his father, and succeeded in reaching the bedside of the sick Secretary, and inflicted a severe cut about the jaw and throat, which was intended to end his life then and there.

I can well recall the circumstances of this sad day to our nation, and the gloom resting over the city of Washington when I arrived there, having been requested to accompany and assist Dr. Gunning in his efforts to bind up Seward's broken jaw.

Lincoln's dead body lay in state, and all our distinguished men were watching the course of events with great anxiety.

Dr. Gunning had been called before the tragedy took place, but did not get get there until after it happened. We visited and treated Mr. Seward in the same room where Mr. Blaine so recently ended his earthly days, making it our dental laboratory.

I will read from Dr. Gunning's report of the case. "The cut inflicted reached from under the right zygoma to the left of the trachea. Steno's duct was severed, and the right fracture laid open externally, the bone being also much exposed in the mouth from the original injury. No arteries had been ligatured. The wound was neatly sewed up, and was healing by first intention, except immediately under the fracture. The swelling and stiffness made the examination difficult, but the ramus proved to be uninjured. There was, however, a second fracture, but on the other side of the mouth, the jaw being fractured on both sides between the bicuspids. The lower jaw contained all the ten forward teeth. The right wisdom tooth and root of the left were all that remained back of the bicuspids. The part in front, containing eight teeth, was drawn down out of place, while the right back fragment, with the wisdom tooth and second bicuspid, was drawn up, showing its fractured end white and bare. Pus discharged profusely from both fractures. The gum was pale and flaccid, in keeping with the general condition of the patient. The upper jaw was entirely without teeth. We deemed it important to set the exposed bone in place as early as possible, and also to give the patient time to recuperate, as he had already been subjected during the morning, not only to a relation of the President's death, but to much that was said and written upon the subject. In the afternoon, while explaining the treatment proper for the case to Dr. Whelan, Dr. Gunning stated his unwillingness to commence, except with the understanding that he should control it entirely.

"*April* 29.—He set the jaw, and held it in place by wire and silk ligatures, took a wax impression of the teeth and gum, and obtained the bite directly from the teeth, etc. Although the front of the jaw containing the eight forward teeth was greatly displaced (before the setting), the silk and wire ligatures held well until May 2, when they were removed and the splint applied. It was of hard, vulcanized rubber, covered the roof of the mouth and adjacent gum, inclosed all the lower teeth, and went down over the gum on the outside somewhat. This splint held the jaw firm for sixty-eight days, when it was removed. There was good union on the left side, but the right fracture was still ununited. For this, however, Dr. Gunning was prepared, as the bone had been exposed so much during the twenty-four days which had elapsed before he set it; and the saliva from the right parotid gland had discharged through the fracture. This was one of the worst features of the case."

Let me only add a word to this.

This case to-day in my hands could be treated without this external arrangement. I should use the simple splint of black rubber, like one I have placed before you, for I feel sure that the artificial denture would act as kindly as the natural teeth.

I have searched all the medical and dental works in vain for any description, either modern or ancient, of any invention prior to these dates, of splints, made of vulcanite, as applied to fractured jaws. It is my privilege to put this on record, as having been personally in charge of the above cases, under Dr. Gunning; and I know what I say when I speak of priority of invention of interdental splints of vulcanite.

In a communication of Dr. T. B. Gunning to the late Professor Agnew, of Philadelphia, wherein he claimed the great superiority of his interdental splints over all other appliances, as doing away with all external bandages, except in the case of an edentulous condition, he remarked that no case without teeth was on record prior to the one reported in May, 1879, in Johnston's "Dental Miscellany," vol. vii., page 63. The case referred to was in my hands for treatment, and, as it was one with many difficulties, treated successfully with poor facilities at command, I will venture to repeat part of the report to you to-night.

"I was called in consultation by Dr. Phinny, of Norwich, Conn., in the case of Asa Bailey, aged seventy years. While recovering from a severe attack of pneumonia he had tried to go down-stairs, but, owing to his feeble condition, fell, and received a compound fracture of the lower jaw, the bone protruding through the gum. His last tooth, a canine on the right side, was knocked out and lost. The fracture was ten days old, with considerable displacement. Dr. Phinny had reduced it with much care and skill, but could not, with bandages, keep the parts in position. There being an entire absence of teeth in the mouth, my first difficulty was to secure the fragments in position. I had never met with such a case. I was without cups or proper conveniences for taking the impressions, and in the country where no dental office was near. Having, however, a little plaster, with a pair of shears and plyers, I made cups for upper and lower jaw out of a tin milk-pan, and succeeded in getting good impressions, from which I made the splint at my office in New York. I did not see the patient again till six days after; but with a little trouble the fractured jaw was placed in the splint. The value of this splint could not be better demonstrated than by the confidence which the patient expressed when it was adjusted. He at once felt that his jaw was secured from the displacing action of the muscles. He could breathe better and swallow with greater ease. I have found this so with all patients under this mode of treatment. I saw the patient again in three weeks, and, on removing the splint, found the fracture well united for so short a time. The patient was then feeling so encouraged that he wished to improve his time by going to work, and before my next visit, three weeks after, he was out following his plough, and doing heavy work with little inconvenience.

"I removed the splint in just six weeks from the day it was first applied. The union was then strong; and it was well ossified in November."

M. Malgaigne, in 1847, says that no case of fractured lower jaw destitute of teeth seems to have been presented for treatment. Such a case seems never to have presented itself to Dr. Gunning in more than forty years' experience.

Books, in great number, have been written on fractures and their treatment, by many of the most learned surgeons of the world,—by Drs. J. F. Malgaigne, Lonsdale, Boyer, Cloquet, Stimson, and William Gibson, who, in 1827, occupied the chair of surgery in the University of Pennsylvania.

The late Frank H. Hamilton, M.D., of this city, in his "Treatise on Fractures and Dislocations," published in Philadelphia in 1863, devotes twenty-two pages to the fracture of the lower jaw; and yet, in all these writings, no mention whatever is made of the interdental splint, until in the 1884 edition he devotes thirty pages to it, and refers particularly to Kingsley's.

Bean, a surgeon in the Confederate service, reported through the *Richmond Medical Journal* that he had over forty cases in the army hospitals. It seems a large number, when we consider how rare the cases are.

Dexter's "Centennial Historical Account of American Dentistry," published in 1876, failed to make any mention of the interdental splints. And yet Dr. Gunning made an exhibit in the Centennial Exposition of the interdental splints for the treatment of fractures of the jaws, including that used in Secretary Seward's case. And in the "Reports and Awards," Group XXIV., edited by Francis A. Walker, chief of the Bureau of Awards, page 25, he says, "He (Dr. Gunning) claims, and I believe justly, that it was the first splint ever used without an appliance outside the mouth." In this same publication fifteen pages are devoted to artificial legs, six pages to trusses, six pages to surgical instruments, three pages to splints, and nineteen pages to dentistry, of which six pages are devoted exclusively to Dr. Gunning and his hard-rubber appliances for fractured jaws and the cleft palate; of such importance did this matter seem to these judges, all of whom were either physicians or surgeons.

One other matter I wish to mention is the use of jaw-cups in which to take impressions, from which to make the interdental splints. They are of particular value to country practitioners, and are very easy to become familiar with. Dr. Gunning's idea was to use gutta-percha in the same cup, after getting the cast, and thus make a splint of it; but I prefer to use vulcanite splints, perforated so that, when they are in place, the tongue and muscles of the cheek are constantly forcing fluids through, and, with a brush, they can be kept very clean.

The important results that surgery and dentistry can accomplish, when worked together in the interest of an unfortunate human being, are well illustrated by the case of Josiah Dutcher, who was under the treatment of Dr. H. B. Sands in 1873, and whose case was reported that year by the New York County Medical Society. I have here the casts made in the Dutcher case and his photograph, which makes him a presentable and even good-looking man after he had been reduced fearfully by the surgeon's knife and the parts restored artificially by the dentist. This was a case of naso-pharyngeal polypi, wherein the surgeon successfully encountered many difficulties by an operation through the mouth, and made extensive removal of parts attended by serious risk of hemorrhage into the glottis.

The time this evening is entirely too short for me to refer in this paper to cases of cleft palates. But I wish to speak of a case of cancerous tumor, as it involved so much where the dentist supplemented the surgeon, supplying lost parts by the use of vulcanite. Of the many cases I have had to treat, I recall one where the gratitude of the patient was expressed to me by letter, within a day or two after the mechanism was placed in his mouth. The late Dr. H. B. Sands had removed a cancerous tumor for the Rev. J. E. Rockwell, of Staten Island, the removal involving one-half of the upper jaw with the hard and soft palate from the median line on the right side. This left him without the power of articulation. The dentures had to be most carefully fitted, and balanced with spiral-springs, he having no natural teeth left; and the upper denture had to do a twofold work, having an obturator attached to it. The patient was so well satisfied that he wrote me he considered he had a new lease of life. He said his voice was good ; in fact, he had just performed a marriage service.

These seven cases of fracture which I have reported, and of which I have shown you the splints, have all been discharged, as cured, in less than four months, with little interruption from every-day business.

I do not suppose you will have in your office to-morrow a simple, compound, double, or edentulous fracture ; they are very rare, as is well shown by the report of one of our largest hospitals for the year ending September 30, 1891. This report shows a total number of two thousand six hundred and thirty-four cases, both medical and surgical; of these, seven were treated for fracture of the lower maxilla and three of the superior maxilla.

In a conversation had within ten days with a surgeon of another large hospital, he stated that he did not believe he had ever had a case of fracture of the lower jaw in his private practice, and had only treated two in hospital practice, in both of which he had used bandages. This will suffice to show the comparative rarity of these cases, and yet we ought certainly to be well prepared to adopt the latest and most approved treatment, when such cases are presented to us.

At times our profession has tried to make a most worthy showing of progress. But, as I said before, the "History of Dental and Oral Science," coming down to 1876, failed to notice this important treatment in dental surgery, and yet it had been in use thirteen years.

The text-books for our dental colleges are faulty, and hence the teaching must be. The great hospitals of this city are treating in the old way with external appliances, as I have myself seen, with regret.

In the Congress which met in London in 1881 there was nothing which helped the dental profession very much, or that would attract the older surgeons to our specialty; and the three other triennial Congresses, which have since met, were not, as far as I know, of any account in improving the method of treating fractures of the maxilla bones, if judged by their published proceedings, which I have looked over carefully.

We are now fast approaching the World's Columbian Dental Congress, where the dental profession will be together in a representative body. At this Congress it is expected that papers and reports of great interest and value will be presented in such numbers and volume as to make books for future dental history.

It is not for me to say that the subject-matter of this paper is worthy a place in, and the consideration of, the Columbian Congress. But I do say that these inventions and devices are equal to any exigencies in the treatment of fractures which can come before us; and some of them have the additional value of being the original inventions used several years before any other splints of vulcanite came into notice. Therefore I suggest that our dental publications shall place them on record. Because they are important in history, as well as practical for present use, they deserve to be on exhibition, and ought not to be crowded out by less important splints which some members of our profession may desire to show with their alleged improvements.

Whoever, in this advanced stage of dental surgery, has to resort to external bandages is not doing for the patient that which tends to his most speedy recovery. But it is he who can so harness the great net-work of muscles which nature has so magnificently formed around the bone as to make them serve in the treatment, which they can do because they pull and draw so kindly and gently over the wounded parts as, by their natural action, to bring the bone into closer unity, thus keeping the bones in place until nature completes the work; he that can do this, I say, is doing a twofold duty by his patient. For he not only repairs the fracture, but he secures a rapid recuperation of his patient by making it convenient to take substantial and nourishing food for the body which is called upon to build up the new bone for the cure.

